# Analysis of Peripapillary Retinal Vessel Diameter in Unilateral Normal-Tension Glaucoma

**DOI:** 10.1155/2017/8519878

**Published:** 2017-06-04

**Authors:** Yong Un Shin, Sang Eon Lee, Heeyoon Cho, Min Ho Kang, Mincheol Seong

**Affiliations:** ^1^Department of Ophthalmology, Hanyang University College of Medicine, Seoul, Republic of Korea; ^2^Department of Ophthalmology, Hanyang University Guri Hospital, Gyeonggi-do, Republic of Korea

## Abstract

**Purpose:**

This study sought to analyze peripapillary retinal vessel diameter and evaluate its correlation with retinal nerve fiber layer (RNFL) thickness in patients with unilateral normal-tension glaucoma (NTG).

**Methods:**

This retrospective study included 37 patients with unilateral NTG and 40 healthy controls. The unilateral NTG patients were selected based on RNFL photography and unilateral visual field (VF) defects from the Humphrey central 30-2 threshold test. The central retinal arteriolar equivalent (CRAE) and central retinal venular equivalent (CRVE) were measured and calculated using retinal photographs and a computer-assisted calculation program. The RNFL thickness was measured using spectral domain optical coherence tomography.

**Results:**

The mean CRAE and CRVE were significantly narrower in the glaucomatous and fellow eyes of the unilateral NTG patients than they were in the normal subjects (*p* < 0.001). There was no significant correlation between CRAE/CRVE and RNFL thickness. There was only a significant correlation between VF severity and RNFL thickness in unilateral NTG eyes.

**Conclusions:**

Both NTG-affected eyes and NTG-fellow eyes in the unilateral NTG patients had narrower central retinal vessel diameters than did the eyes of normal subjects. Our results show that vascular factors may play a role in the NTG pathogenesis.

## 1. Introduction

Normal-tension glaucoma (NTG) is a chronic optic neuropathy characterized by progressive injury to the retinal ganglion cells (RGCs), which subsequently leads to visual field loss and is parallel to primary open-angle glaucoma (POAG) with the exception of a normal intraocular pressure (IOP) [1]. The pathogenesis of NTG is multifactorial but remains poorly understood. Unlike POAG, vascular theories are considered to be a significant pathogenesis of NTG [2]. The vascular theory describes abnormal perfusion of the optic nerve for various reasons (both systemic and ocular reasons), which leads to the development of NTG [3–6].

Among those who support the vascular theory of glaucoma, there has been some interest in the diameter of the retinal vessels [7–9]. Jonas found that the parapapillary retinal vessel diameters were correlated with optic disc structural damage of patients with glaucoma [7]. Rader et al. found that the general narrowing of the caliber of retinal arteries was related to the severity of optic nerve damage in eyes with classic glaucoma and NTG [8]. Mitchell et al. also showed that eyes with open-angle glaucoma had a significantly narrower retinal arteriolar diameter than did normal eyes [9].

Open-angle glaucoma, including NTG, is mostly commonly bilateral. However, unilateral NTG is also observed in the clinical setting. Several authors have reported that the (presumably) normal fellow eyes in cases of unilateral glaucoma sometimes also demonstrate structural changes such as decreased retinal nerve fiber layer (RNFL) thickness; these fellow eyes can eventually progress to glaucoma [10–12]. However, there are no prior studies that have studied retinal vessel diameter in patients with unilateral NTG.

Therefore, we used a computer-assisted method to compare retinal vessel diameter among affected and unaffected fellow eyes in patients with unilateral NTG and in a normal control group. We also evaluated the relationships between retinal vessel diameter and other factors, such as retinal nerve fiber layer (RNFL) thickness and IOP.

## 2. Methods

### 2.1. Study Subjects

We retrospectively reviewed the medical records of 47 patients with unilateral NTG (47 glaucomatous eyes and 47 fellow eyes) and age- and sex-matched healthy controls (40 right eyes). All of the included patients were examined at the Department of Ophthalmology at Hanyang University Guri Hospital between March 2012 and November 2015. This study design was reviewed and approved by the Institutional Review Board of Hanyang University Guri Hospital. All participants underwent detailed eye examination at the first visit, including Goldmann applanation tonometry, ultrasonic pachymetry (SP-200, Tomey, Nagoya, Japan), Humphrey central 30-2 threshold testing (Zeiss-Humphrey, San Leandro, CA, USA), stereoscopic photography after pupil dilation (VX-10 fundus camera, Kowa), and retinal nerve fiber layer thickness (RNFL) using spectral domain optical coherence tomography (SD-OCT, 3D-OCT 2000, Topcon, Tokyo, Japan) in both eyes. Before the disc photography exam, patients waited for about 30 minutes while sitting comfortably and then underwent optic disc photography of both eyes in a sitting position.

Diagnosis of NTG was based on both the presence of typical glaucomatous changes in the optic disc and glaucomatous visual field (VF) defect. Glaucomatous optic disc change included increased cupping (vertical cup-disc ratio > 0.7), a difference in the vertical cup-disc ratio > 0.2 between the two eyes, diffuse or focal neural rim thinning, disc hemorrhage, or RNFL defects. Eyes defined as having glaucomatous VF defects met two of the following three criteria: (1) a cluster of three points with a probability of <5% on a pattern deviation map in at least one hemifield, including at least one point with a probability of <1% or a cluster of two points with a probability of <1%; (2) a glaucoma hemifield test result outside the normal limit; and (3) a pattern standard deviation < 5%. Reliable VF assessment was defined as a VF test with a false-positive error < 15%, a false-negative error < 15%, and a fixation loss < 20%. In addition, the IOP measured by Goldmann applanation tonometry was consistently <21 mmHg. We excluded angle closure glaucoma and secondary glaucoma. Unilateral NTG was defined when NTG was presented in only one eye with the fellow being free of any damage. We only included patients with glaucoma-specific optic disc changes and corresponding RNFL and visual field changes and who were diagnosed with NTG for the first time in our clinic.

The exclusion criteria were as follows: the presence of one or any systemic vascular diseases such as hypertension, diabetes, myocardial ischemia, or stroke; use of one or any systemic medications; a history of glaucoma-related medications or intraocular surgery; high refractive error (spherical equivalent > ±6 diopters); and poor image quality of fundus photography. In addition, if patients had abnormal color vision, any neurological symptoms, a history of acute visual disturbance, nonglaucomatous visual field defect (e.g., visual field defect respecting the vertical meridian, central, or cecocentral visual field defect), and the presence of optic disc pallor, they were excluded from this study.

### 2.2. Retinal Vessel Caliber Measurement

**Figure 1:**
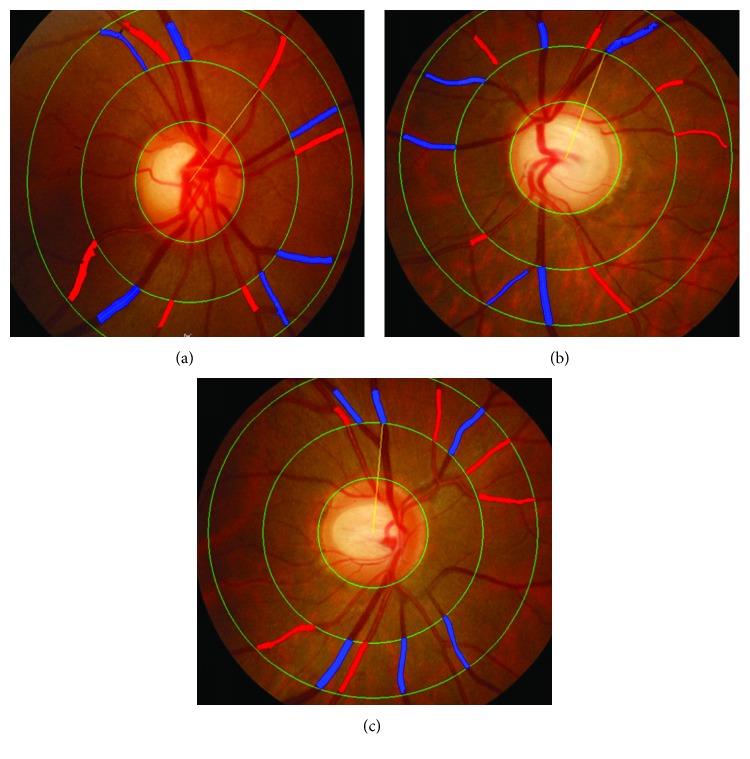
Examples of digitized retinal photographs measuring retinal vessel diameters. (a) Central retinal arteriolar equivalent (CRAE) (181.40 *μ*m) and central retinal venular equivalent (CRVE) (226.77 *μ*m) in the control group. (b) CRAE (153.36 *μ*m) and CRVE (202.34 *μ*m) in the normal-tension glaucoma (NTG) eye. (c) CRAE (167.56 *μ*m) and CRVE (207.02 *μ*m) in the NTG-fellow eye. The CRAE and CRVE were narrower in the glaucomatous eye and their fellow eyes in unilateral NTG patients than they were in the normal eyes.

We measured the retinal vessel diameters using a previously described semiautomated computer-assisted method (IVAN, University of Wisconsin-Madison; Ferrier NJ) [13, 14]. Two researchers (YUS and SEL) performed this process. In brief, the photographs were projected onto a 21-inch computer monitor. We measured all of the vessels passing completely through a circumferential zone of 0.5–1 disc diameter from the optic disc margin. The grader identified each vessel as an arteriole or a venule using the original photograph for reference. The central width calculates the average width from 5 equidistant measures of each vessel or branch (in micrometers) (Figure 1). The Parr-Hubbard formula was used to standardize the arteriolar and venular calibers of each eye, which were summarized as the central retinal arteriolar equivalent (CRAE) or central retinal venular equivalent (CRVE), respectively [15]. The width of the 6 largest arterioles and 6 largest venules are required to calculate the CRAE and CRVE, respectively. All values measured by the two readers were averaged for image analysis. The CRAE was divided by the CRVE to obtain the arteriole-to-venule ratio (AVR). An AVR of 1.0 suggests that arteriolar diameters were, on average, the same as venular diameters in that eye. In contrast, a smaller AVR suggests narrower arterioles than venules.

Statistical analyses were performed using SPSS version 21.0 (SPSS Inc., Chicago, IL, USA). Interobserver repeatability was examined by calculating the interclass correlation (ICC). We divided subjects into three groups as follows: NTG-affected eyes, NTG-fellow eyes, and control eyes. The outcome variables were compared among the three groups using the ANOVA test. Linear regression analysis was used to measure potential correlations between factors such as age, IOP, refractive errors, mean deviation (MD), pattern standard deviation (PSD), CRAE, CRVE, and RNFL thickness. The data are presented as mean ± standard deviation (SD). In all statistical analyses, *p* < 0.05 was considered statistically significant.

## 3. Results

We enrolled 37 subjects with unilateral NTG and 40 healthy subjects who met the study criteria. Ten patients were excluded due to concomitant systemic vascular diseases (4 eyes), low quality of fundus photography (5 eyes), or high refractive error (1 eye). The baseline clinical characteristics of the two groups are shown in Table 1. The mean age was 55.8 ± 10.7 years in the unilateral NTG group and 57.0 ± 9.1 years in the control group (*p* = 0.995). Men made up 45.9% of the unilateral NTG group and 45% of the control group (*p* = 0.837).

We compared various ocular variables among the three groups (NTG-affected eyes, NTG-fellow eyes, and control eyes). The mean IOP and spherical equivalents did not show any significant difference among the groups (*P* = 0.532 and 0.229, resp.). Mean central corneal thickness was 528.7 ± 29.5 (range 498–597) *μ*m in the NTG-affected eyes, 529.6 ± 29.1 (range 500–595) *μ*m, and 540.1 ± 37.6 (range 460–586) *μ*m in normal control eyes, but no statistical differences were observed among groups (*p* = 0.591). In the visual field test, the MD was significantly worse in the NTG-affected eyes (−4.7 ± 4.3) than it was in the NTG-fellow eyes (−1.3 ± 1.6) or normal control group (−0.5 ± 1.1) (*p* < 0.001). The mean PSD was also significantly worse in the NTG-affected eyes (6.2 ± 3.8) than it was in the NTG-fellow eyes (2.0 ± 0.8) and normal control group (1.7 ± 0.3) (*p* < 0.001). In most quadrants, with exception of the nasal quadrant, RNFL thickness was significantly thinner in glaucomatous eyes than it was in fellow eyes and normal eyes (*p* < 0.05). There was no significant difference in RNFL thickness between the NTG-fellow eyes and control eyes (*p* > 0.05 in all quadrants).

**Figure 2:**
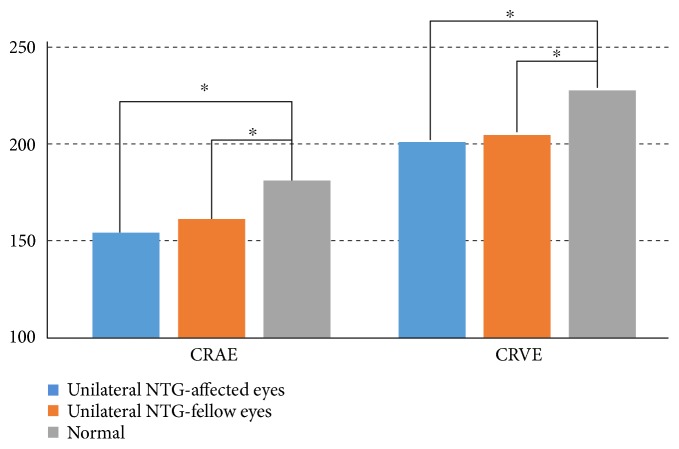
Comparison of the retinal vessel diameters among the three groups. Both central retinal arteriolar equivalent (CRAE) and central retinal venular equivalent (CRVE) were significantly narrower in the normal-tension glaucoma (NTG) eyes and fellow eyes than they were in the normal eyes (asterisks indicate *p* < 0.001, ANOVA, post hoc Tukey's test).

There were no significant differences between the groups with regard to retinal vessel diameter, as measured by two researchers using IVAN (*r* > 0.8 for all variables, ICC). The CRAE was significantly narrower in the NTG-affected eyes (154.6 ± 15.8 *μ*m) and the NTG-fellow eyes (161.2 ± 18.7 *μ*m) than it was in the normal control group (181.7 ± 10.5 *μ*m) (*p* < 0.001, both). The CRVE was significantly narrower in the NTG-affected eyes (200.8 ± 22.8 *μ*m) and the NTG-fellow eyes (205.0 ± 22.6 *μ*m) than it was in the normal control group (228.2 ± 13.2 *μ*m) (*p* < 0.001, both). There were no significant differences in AVR across all three groups (*p* = 0.198). The CRAE and CRVE were narrower in NTG-affected eyes than they were in NTG-fellow eyes, although the difference was not statistically significant (Figure 2).

Univariate linear regression analysis was used to evaluate the associations between measured RNFL thickness and age, IOP, RE, MD, PSD, CRAE, and CRVE among the three groups. There was a significant correlation between VF severity (MD, PSD) and RNFL thickness in unilateral NTG-affected eyes (*p* = 0.002 in MD and 0.039 in PSD). However, there were no other significant correlations. These data are shown in Table 2.

## 4. Discussion

Our study demonstrates that the retinal vessel calibers around the optic disc (both arteries and veins) were significantly narrower in NTG-affected eyes than they were in normal control eyes. In addition, both retinal arterioles and venules were significantly narrower in NTG-fellow eyes than they were in normal control eyes.

Several prior studies have identified associations between retinal vessel caliber and the presence of glaucoma. Lee et al. found that generalized narrowing of the retinal vessel diameter was associated with more advanced glaucoma stage, increasing visual field defects, and increased peripapillary atrophy in primary open-angle glaucoma (POAG) [16]. The study by Chang et al. enrolled patients with bilateral NTG; one eye was randomly selected from each subject for measuring retinal vessel diameters and comparing them with those of normal controls. They suggested that smaller central retinal vessel diameters were observed in patients with bilateral NTG compared with normal controls, which is consistent with our results. In contrast, we enrolled only unilateral NTG patients in this study and compared retinal vessel diameters among NTG-affected eyes, NTG-fellow eyes, and normal control eyes, finding smaller retinal vessels in NTG-fellow eyes than in normal control eyes [17]. In eyes with POAG, Hall et al. found a strong association between decreased peripapillary arteriole diameter and visual field defects in the corresponding hemifield [18]. Rankin et al. reported a higher prevalence of focal arteriolar narrowing in patients with chronic open-angle glaucoma and NTG than in patients with ocular hypertension and normal controls [19]. In addition, the Blue Mountains Eye Study found that eyes with glaucomatous damage had significantly smaller mean retinal vessel diameters and were at least twice as likely to have generalized arteriolar narrowing as were normal eyes [9]. Our results are consistent with those of previous reports regarding vascular diameter in NTG patients. In contrast, Rao et al. found no significant difference in the vessel diameter of affected quadrants compared with that of unaffected quadrants in the same eye or in age- and severity-matched NTG or POAG controls with hemifield damage [20].

To the best of our knowledge, no prior studies have evaluated vascular diameter in patients with unilateral NTG. An interesting finding of our study is that both CRAE and CRVE were significantly narrower in the fellow eyes of unilateral NTG than they were in normal control eyes. We hypothesized that the fellow eyes of unilateral NTG are also affected by insufficient blood supply, resulting in glaucomatous optic damage. Previous researches have suggested that the fellow eyes of unilateral glaucoma also accumulate glaucomatous change. Armaly found significant differences in the appearance of the optic disc of the damaged and undamaged eyes of patients with unilateral visual field defects; however, the cups in the undamaged fellow eyes were significantly larger than those in normal eyes. These larger cups were thought to indicate either a greater susceptibility to field loss in eyes with a genetically large cup or acquired tissue changes of the optic nerve head prior to the development of field defects in the fellow eye [10]. Nicolela et al. found that eyes with normal visual fields in patients with asymmetric glaucoma had reduced blood flow in their retrobulbar vessels using color-doppler imaging [21]. Using SD-OCT, Liu et al. found RNFL thickness loss in a substantial number of contralateral eyes of patients with unilateral glaucoma [11]. Kim et al. found that the anterior prelaminar depth was significantly greater and the prelaminar tissue was significantly thinner in the contralateral eyes of patients with unilateral glaucoma compared to those of healthy control eyes [12]. These findings suggest that subclinical or preperimetric changes of the lamina cribrosa and/or prelaminar tissue are already present in the contralateral eyes of unilateral glaucoma.

Some reports have suggested that there is a relationship between RNFL thickness and retinal vessel diameters. However, this hypothesis is controversial. Zheng et al. found a significant association between narrower retinal vessel caliber and RNFL thinning in a population-based study of Asian Malays 40–80 years old without glaucoma; however, there was no such relationship in patients with glaucoma [22]. Chang et al. found a significant correlation between RNFL thickness and CRAE in NTG patients [17]. Kim et al. reported that the mean diameter of the temporal retinal vessels in the quadrants with RNFL defects was significantly smaller in patients with NTG than in quadrants without RNFL defects. In addition, the temporal retinal arteriolar diameter was correlated with RNFL defects and RNFL thickness [23]. We did not find any significant correlation between vessel diameters and RNFL thickness in the unilateral glaucoma group. This result may be explained by the large proportion of patients with early glaucoma enrolled in this study. Although we included patients with all stages of disease, most of the enrolled subjects with unilateral NTG had early glaucoma (26 eyes in 37 NTG patients, 70%). Further studies with patients of varying severity of disease are warranted.

This study had several limitations. First, the retrospective and cross-sectional design with small sample sizes makes it difficult to explain the causality between retinal vessel diameters and NTG development. Second, systemic vascular diseases such as hypertension, diabetes mellitus, and stroke may have influenced our results. Although we excluded patients with systemic vascular diseases based on interview, we may have missed some underlying disease, as we did not perform detailed cardiovascular exams. In addition, we did not measure blood pressure, which may influence the retinal vessel diameter at the time of ophthalmologic exams. Third, although we used a computer-assisted semiautomated method to measure retinal vessel diameter from digitized fundus photographs, we sometimes adjusted the vessel diameter manually. Therefore, we may have introduced human error when adjusting the ill-definite vessels manually.

In conclusion, our results suggest that the normal fellow eyes in unilateral NTG also have peripapillary retinal vascular narrowing. In a pathogenic point of view, retinal vascular changes may precede definite glaucomatous change in NTG, which supports vascular mechanism of NTG. Clinically, unaffected contralateral eyes in unilateral NTG should be examined regularly to detect whether definite glaucomatous change is present or not. Further prospective investigation is needed to determine whether such vascular changes reflect an ischemic pathologic process, ultimately leading to the loss of retinal ganglion cells.

## Figures and Tables

**Table 1:** Comparison of variables among glaucomatous eyes, fellow eyes, and control eyes.

	Unilateral NTG	Unilateral NTG	Control eye (3)	*p* value	Post hoc^c^		
	Affected eye (1)	Fellow eye (2)			1 versus 2	2 versus 3	1 versus 3
Sex (M : F)	17 : 20		18 : 22	0.995^a^			
Age (yrs)	55.8 ± 10.7		57.0 ± 9.1	0.837^b^			
IOP (mmHg)	15.1 ± 2.6	14.6 ± 2.7	15.4 ± 3.7	0.532^b^			
Central corneal thickness (*μ*m)	528.7 ± 29.5	529.6 ± 29.1	540.1 ± 37.6	0.591^b^			
Spherical equivalent (diopter)	−1.0 ± 1.8	−0.9 ± 2.0	−0.3 ± 1.6	0.229^b^			
MD	−4.7 ± 4.3	−1.3 ± 1.6	−0.5 ± 1.1	<0.001^b^	<0.001	0.365	<0.001
PSD	6.2 ± 3.8	2.0 ± 0.8	1.7 ± 0.3	<0.001^b^	<0.001	0.847	<0.001
Total RNFL thickness (*μ*m)	88.6 ± 12.9	100.5 ± 8.2	102.1 ± 11.4	<0.001^b^	<0.001	0.810	<0.001
Superior (*μ*m)	102.9 ± 19.6	118.6 ± 11.1	118.8 ± 15.0	<0.001^b^	<0.001	0.997	<0.001
Temporal (*μ*m)	70.2 ± 14.2	77.8 ± 9.3	77.2 ± 12.9	0.015^b^	0.026	0.975	0.040
Inferior (*μ*m)	103.8 ± 21.6	125.8 ± 13.7	127.4 ± 15.6	<0.001^b^	<0.001	0.908	<0.001
Nasal (*μ*m)	77.6 ± 16.0	80.4 ± 14.3	85.3 ± 16.1	0.088^b^			
CRAE (*μ*m)	154.6 ± 15.8	161.2 ± 18.7	181.7 ± 10.5	<0.001^b^	0.154	<0.001	<0.001
CRVE (*μ*m)	200.8 ± 22.8	205.0 ± 22.6	228.2 ± 13.2	<0.001^b^	0.635	<0.001	<0.001
AVR	0.77 ± 0.06	0.78 ± 0.06	0.80 ± 0.05	0.198^b^			
Total (*n*)	37	37	40				

Data are presented as mean ± standard deviation. NTG: normal-tension glaucoma; IOP: intraocular pressure; MD: mean deviation; PSD: pattern standard deviation; RNFL: retinal nerve fiber layer; CRAE: central retinal arteriole equivalent; CRVE: central retinal venule equivalent; AVR: arteriovenous ratio. ^a^Pearson's chi-square test. ^b^Analysis of variance (ANOVA) test. ^c^Post hoc Tukey's test.

**Table 2:** Univariate linear regression analysis showing the associations between retinal nerve fiber layer thickness and other variables in glaucomatous eyes, fellow eyes, and control eyes.

	Unilateral NTG		Unilateral NTG		Control eyes	
	Affected eyes		Fellow eyes			
	Regression coefficient	*p* value	Regression coefficient	*p* value	Regression coefficient	*p* value
Age (years)	−0.066	0.699	−0.057	0.738	−0.143	0.380
IOP (mmHg)	0.174	0.303	0.057	0.737	−0.148	0.362
Spherical equivalent (diopter)	0.112	0.507	0.038	0.821	0.093	0.569
MD	0.483	**0.002**	0.110	0.515	0.065	0.691
PSD	−0.341	**0.039**	−0.182	0.280	−0.267	0.095
CRAE (*μ*m)	−0.05	0.770	0.197	0.242	0.216	0.181
CRVE (*μ*m)	−0.006	0.972	−0.061	0.721	0.260	0.105

NTG: normal-tension glaucoma; IOP: intraocular pressure; MD: mean deviation; PSD: pattern standard deviation; CRAE: central retinal arteriole equivalent; CRVE: central retinal venule equivalent.
